# Efficient Transformation of Oil Palm Protoplasts by PEG-Mediated Transfection and DNA Microinjection

**DOI:** 10.1371/journal.pone.0096831

**Published:** 2014-05-12

**Authors:** Mat Yunus Abdul Masani, Gundula A. Noll, Ghulam Kadir Ahmad Parveez, Ravigadevi Sambanthamurthi, Dirk Prüfer

**Affiliations:** 1 Advanced Biotechnology and Breeding Centre, Malaysian Palm Oil Board (MPOB), Kuala Lumpur, Malaysia; 2 Westfälische Wilhelms-Universität Münster, Institut für Biologie und Biotechnologie der Pflanzen, Münster, Germany; 3 Fraunhofer Institut für Molekularbiologie und Angewandte Ökologie, Münster, Germany; Wuhan University, China

## Abstract

**Background:**

Genetic engineering remains a major challenge in oil palm (*Elaeis guineensis*) because particle bombardment and *Agrobacterium*-mediated transformation are laborious and/or inefficient in this species, often producing chimeric plants and escapes. Protoplasts are beneficial as a starting material for genetic engineering because they are totipotent, and chimeras are avoided by regenerating transgenic plants from single cells. Novel approaches for the transformation of oil palm protoplasts could therefore offer a new and efficient strategy for the development of transgenic oil palm plants.

**Methodology/Principal Findings:**

We recently achieved the regeneration of healthy and fertile oil palms from protoplasts. Therefore, we focused on the development of a reliable PEG-mediated transformation protocol for oil palm protoplasts by establishing and validating optimal heat shock conditions, concentrations of DNA, PEG and magnesium chloride, and the transfection procedure. We also investigated the transformation of oil palm protoplasts by DNA microinjection and successfully regenerated transgenic microcalli expressing green fluorescent protein as a visible marker to determine the efficiency of transformation.

**Conclusions/Significance:**

We have established the first successful protocols for the transformation of oil palm protoplasts by PEG-mediated transfection and DNA microinjection. These novel protocols allow the rapid and efficient generation of non-chimeric transgenic callus and represent a significant milestone in the use of protoplasts as a starting material for the development of genetically-engineered oil palm plants.

## Introduction

The oil palm genetic engineering program was initiated by the Malaysian Palm Oil Board (MPOB), then known as the Palm Oil Research Institute of Malaysia (PORIM), in the early 1990s [1]. The main objectives of this program are to produce transgenic oil palm (*Elaeis guineensis*) with a higher content of oleic acid, modified oil quality (e.g. a higher content of stearic acid), and the ability to produce value-added oils such as palmitoleic and ricinoleic acid, as well as novel products such a biodegradable plastics. It has been suggested that such targets could be achieved 80% more rapidly by combining genetic engineering and tissue culture techniques [2]. In addition, oil palm is a perennial crop and high-value products could be produced continuously for at least 30 years, making this species an ideal candidate for genetic engineering.

Particle bombardment and *Agrobacterium*-mediated transformation can be used to introduce genes into oil palm, and stable transformation has been achieved using both methods. Successful particle bombardment requires the establishment of optimal physical and biological parameters during transformation and the use of appropriate selectable markers and promoters [3]. A biolistic protocol for the production of glufosinate-resistant transgenic oil palm has been developed [4], and in light of its success thousands of embryogenic calli have been bombarded with genes involved in fatty acid biosynthesis to increase the accumulation of oleic acid [5], [6], [7], stearic acid [8], polyhydroxybutyrate (PHB) and polyhydroxyvalerate (PHBV) [9], [10], [11].

One drawback of particle bombardment is that it often promotes the integration of multiple transgene copies [12], whereas *Agrobacterium*-mediated transformation is more likely to introduce either single-copy or low-copy-number transgenes, as shown e.g. in rice [13] and maize [14]. However, *Agrobacterium*-mediated oil palm transformation is inefficient because oil palm is a monocotyledonous species outside the normal *Agrobacterium tumefaciens* host range. Nevertheless, there have been many attempts to improve transformation efficiency, e.g. by using immature oil palm embryos as the target tissue for particle bombardment and *Agrobacterium*-mediated transformation [15], [16], and by optimizing other transformation parameters [17]. This led to the development of insect-resistant transgenic oil palms expressing *Bacillus thuringiensis* (Bt) insecticidal proteins [18] and cowpea trypsin inhibitor (CpTI) [19].

The studies described above revealed that 3–5 years are required to generate transgenic oil palm plants by particle bombardment or *Agrobacterium*-mediated transformation and that the process is highly inefficient. The frequency of escapes and chimeric plants is high because the long selection process during callus formation and somatic embryogenesis encourages the growth of non-transformed cells. It is possible that the optimization of DNA delivery and selection could overcome such challenges but an alternative approach is to use protoplasts as transformation targets because they are totipotent, and chimeras can thus be avoided by regenerating transgenic plants from single cells. Novel approaches for the transformation of oil palm protoplasts could therefore offer a new and efficient strategy for the development of transgenic oil palm plants. As well as particle bombardment and *Agrobacterium*-mediated transformation, protoplasts can also be transformed using polyethylene glycol (PEG), electroporation or DNA microinjection.

Recently, we established an efficient protocol for the preparation of oil palm protoplasts and the regeneration of healthy and fertile oil palm plants [20]. Here we developed novel transformation protocols based on PEG-mediated transfection and DNA microinjection showing that protoplasts are suitable as a target for oil palm genetic engineering. We successfully expressed a reporter gene encoding green fluorescent protein (GFP) allowing the rapid and efficient generation of non-chimeric transgnic callus without the use of standard selectable markers. Our results represent a significant milestone in development of genetically-engineered oil palm plants.

## Results

### PEG-mediated transfection of oil palm protoplasts

#### Choice of protoplast source.

**Figure 1:**
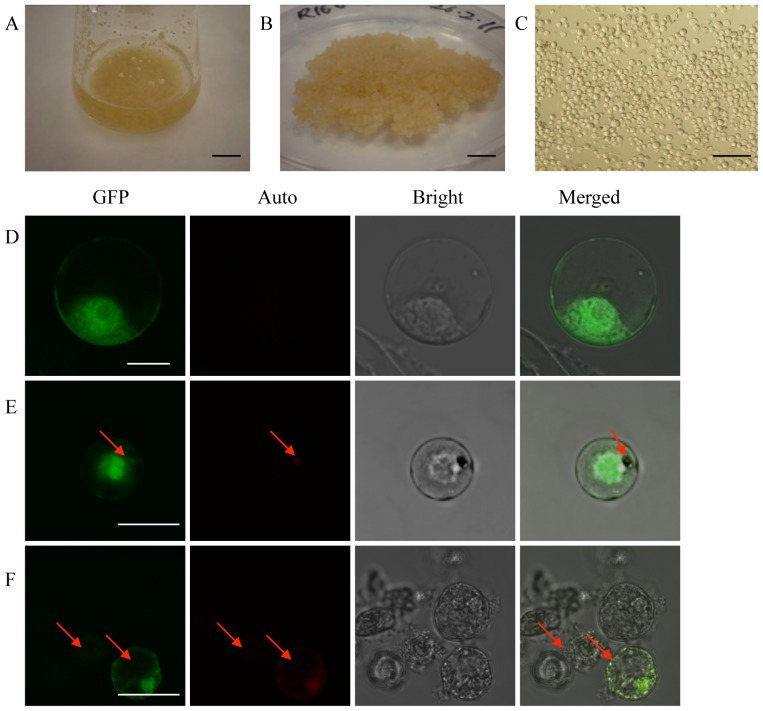
Oil palm protoplasts showing GFP fluorescence. A 3-month-old oil palm cell suspension culture in Y35N5D2iP liquid medium (A) was collected and cultured on Y35N5D2iP solid medium (B) for protoplast isolation (C). Transient GFP fluorescence was observed in protoplasts isolated from the 3-month-old cell suspension culture after subculture for 7 days (D) and 14 days (E), and protoplasts isolated from the 4-month-old cell suspension culture (F). CLSM images are shown representing GFP fluorescence (GFP), autofluorescence (Auto) and bright field (Bright) as well as three-layer images (Merged) of the protoplasts. Red arrows indicate autofluorescence. Scale bar  = 1 cm in (A) and (B), 100 µm in (C), 10 µm in (D), 25 µm in (E) and (F).

In order to identify the most suitable protoplasts for PEG-mediated transformation, we tested protoplasts from different sources, namely those isolated 7 or 14 days after the subculture of a cell suspension culture that had been cultivated for either 3 or 4 months (Figure 1A–C). For the initial protoplast transfection experiments we used 10 µg of CFDV-hrGFP plasmid DNA mixed with 40% (w/v) PEG dissolved in Rinse solution, and incubated the protoplasts for 10 min. The appearance of fluorescent protoplasts 72 h later indicated that the hrGFP gene was expressed successfully in protoplasts from all the sources we tested (Figure 1D–F). However, the transfection efficiency was low (<0.1%) because most of the protoplasts were severely damaged and only a small number of fluorescent protoplasts survived. GFP fluorescence was distributed throughout the cytoplasm and nucleus, extending to the plasma membrane in protoplasts from both the 7 and 14 day subcultures (Figures 1D–F). Protoplasts from the 14-day subcultures also showed pale yellow autofluorescence, which was more intense in the protoplasts derived from the 4-month-old cell suspension culture (Figures 1E–F). We selected protoplasts isolated from the 7-day subculture of the 3-month-old suspension culture as the most suitable substrates for PEG-mediated transformation because of the lack of autofluorescence, thus reducing the likelihood of false positive results.

#### MgCl_2_ concentration and DNA incubation period

**Figure 2:**
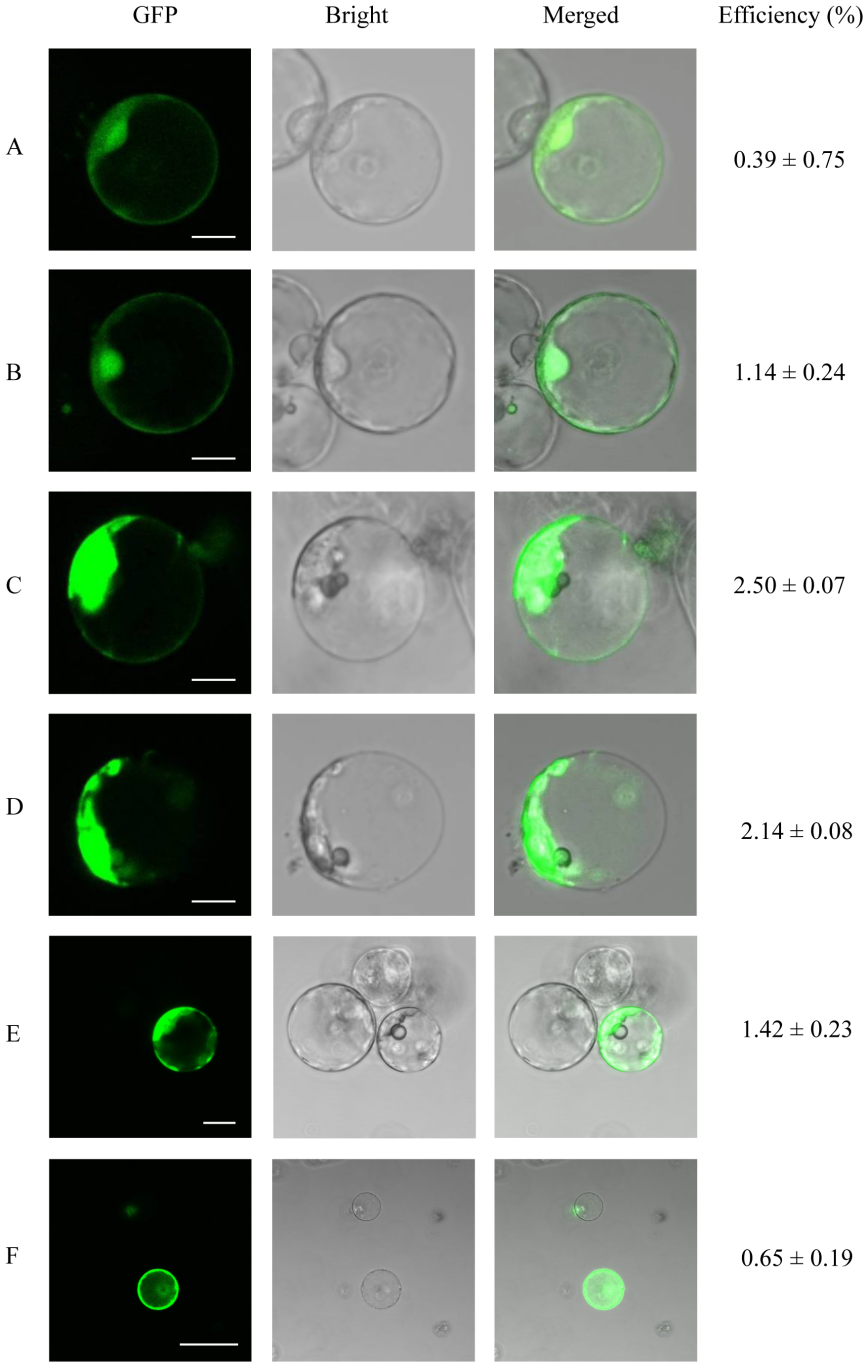
Transfection efficiency is affected by different concentrations of MgCl_2_ and the DNA incubation time. Oil palm protoplasts were transfected with 10 µg CFDV-hrGFP plasmid using 40% (w/v) PEG solution with MgCl_2_ at concentrations of 10 mM (A), 25 mM (B), 50 mM (C) and 100 mM (D). Oil palm protoplasts were incubated with 10 µg CFDV-hrGFP plasmid DNA for 15 min (E) or 30 min (F), and then mixed with PEG-MgCl_2_ solution. Transfection efficiency was calculated as the number of GFP-fluorescent protoplasts divided by the total number of protoplasts in one representative microscope field. The transfection efficiencies represent the mean of three replicates. Scale bar  = 10 µm in (A)–(E), 75 µm in (F).

We investigated the impact of Mg^2+^ ions on transfection efficiency by incubating oil palm protoplasts as above for 10 min in the presence of 10 µg of CFDV-hrGFP plasmid DNA mixed with 40% (w/v) PEG dissolved in Rinse solution, but this time we varied the concentration of Mg^2+^ ions by preparing solutions containing 10 mM (Figure 2A), 25 mM (Figure 2B), 50 mM (Figure 2C) and 100 mM MgCl_2_ (Figure 2D). The presence of 10 mM MgCl_2_ increased the transfection efficiency by four-fold to 0.39% (Figure 2A) compared to a PEG solution lacking magnesium (<0.1%, data not shown) but higher concentrations were even more beneficial, and the greatest efficiency (2.5%) was achieved in the presence of 50 mM MgCl_2_ (Figure 2B–D). GFP fluorescence was more intense in the protoplasts transfected at higher Mg^2+^ concentrations, indicating the more efficient uptake of exogenous DNA (Figure 2A–D).

Having established the optimal Mg^2+^ concentration for transfection, we next varied the incubation time following the addition of plasmid DNA but prior to the addition of the PEG/MgCl_2_ solution (Figure 2E–F). Prolonging the incubation period to 15 min (Figure 2E) or 30 min (Figure 2F) reduced the transfection efficiency to 1.42% and 0.65%, respectively. Therefore we reverted to the original incubation period of 10 min.

#### Concentrations of DNA and PEG, and heat shock treatment

**Figure 3:**
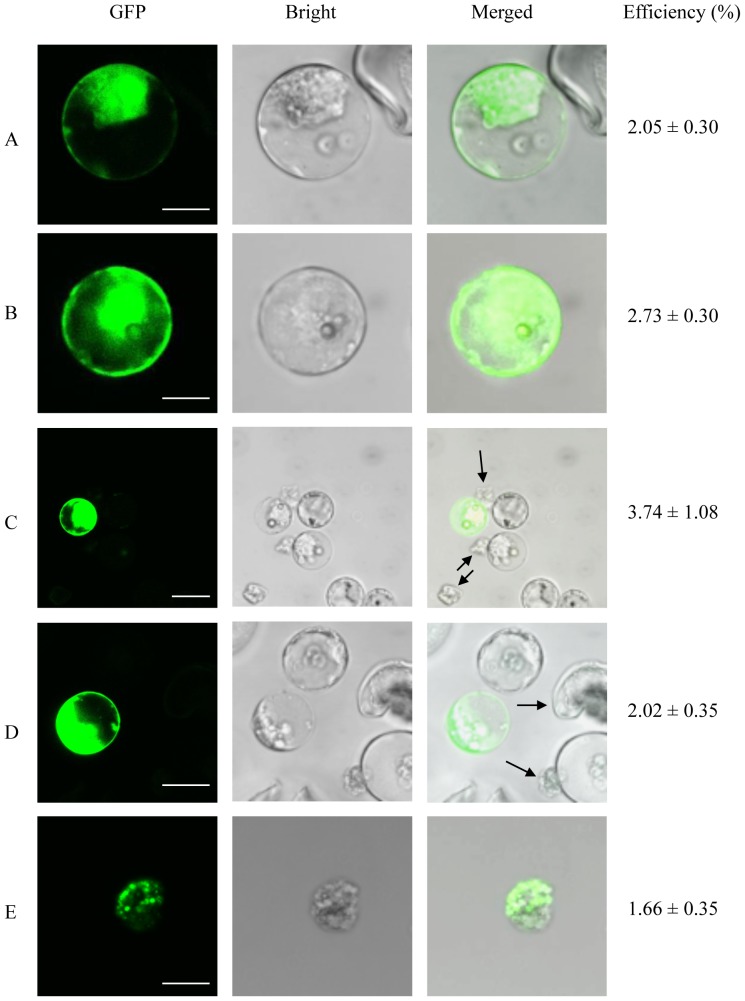
Effects of DNA and PEG concentrations on transfection efficiency. Oil palm protoplasts were transfected with 25 µg (A) or 50 µg (B) of CFDV-hrGFP plasmid DNA, and with 50 µg CFDV-hrGFP plasmid DNA in the presence of 25% (C), 40% (D) or 50% PEG (E). Black arrows indicate damaged protoplasts caused by PEG toxicity. The transfection efficiencies represent the mean of three replicates. Scale bar  = 10 µm in (A) and (B), 25 µm in (C)–(F).

Next we investigated the impact of DNA concentration on transfection efficiency by incubating protoplasts in the presence of 25 µg (Figure 3A) or 50 µg (Figure 3B) of CFDV-hrGFP plasmid DNA using the 40% (w/v) PEG/50 mM MgCl_2_ solution discussed above. High transfection efficiencies were achieved in both cases, but the lower DNA concentration was less efficient (2.05%, Figure 3A) than the original 50-µg dose (2.73%, Figure 3B). The GFP fluorescence was also more intense in protoplasts transfected with higher concentrations of DNA probably because more was taken up into the cell.

We also investigated the effects of different PEG concentrations, varying the (w/v) concentration of PEG 4000 from 25% (Figure 3C), to 40% (Figure 3D) and also 50% (Figure 3E). In each case, the different PEG concentrations were tested with the optimal DNA and MgCl_2_ concentrations and 10-min DNA incubation time established above. The corresponding transfection efficiencies were 3.74%, 2.02% and 1.66%, showing that 25% (w/v) PEG is optimal for the transformation of oil palm protoplasts. There was no difference in terms of GFP fluorescence regardless of the PEG concentration, suggesting that PEG does not affect hrGFP gene expression but may instead affect the viability of the oil palm protoplasts at concentrations higher than 25%.

**Figure 4:**
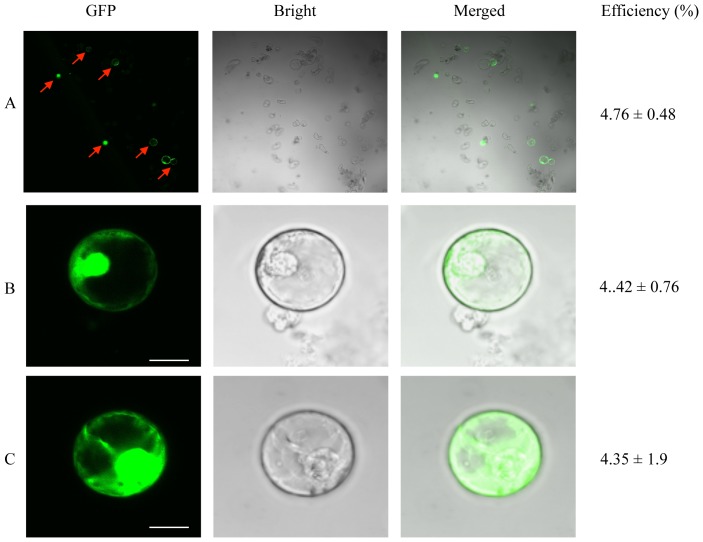
Effect of heat shock treatment on transfection efficiency. Oil palm protoplasts were incubated at 45°C for 5 min and then on ice for 1 min before mixing with 50 µg of CFDV-hrGFP plasmid DNA and then PEG-MgCl_2_ solution (25% PEG, 50 mM MgCl_2_, 3% KCl and 3.6% mannitol, pH 6.0). The protoplasts were incubated at 26°C for 3 days (A), 6 days (B) and 9 days (C). Red arrows indicate protoplasts showing GFP fluorescence. The transfection efficiencies represent the mean of three replicates. Scale bar  = 100 µm in (A), 10 µm in (B) and (C).

Finally, we investigated the effect of heat shock treatment by incubating the protoplasts at 45°C for 5 min and then cooling on ice for 1 min before adding 50 µg of CFDV-hrGFP plasmid DNA, incubating for 10 min as above and then adding 25% (w/v) PEG in 50 mM MgCl_2_. This treatment increased the transfection efficiency even further to 4.76% (Figure 4A) indicating that a heat shock significantly improves DNA uptake. Fluorescent protoplasts were observed continuously for 9 days indicating that hrGFP fluorescence remains stable following transfection, although the frequency declined over time from 4.42% on day 6 (Figure 4B) to 4.35% on day 9 (Figure 4C).

### Transformation of oil palm protoplasts by DNA microinjection

#### Choice of protoplast platform and optimal injection time.

**Figure 5:**
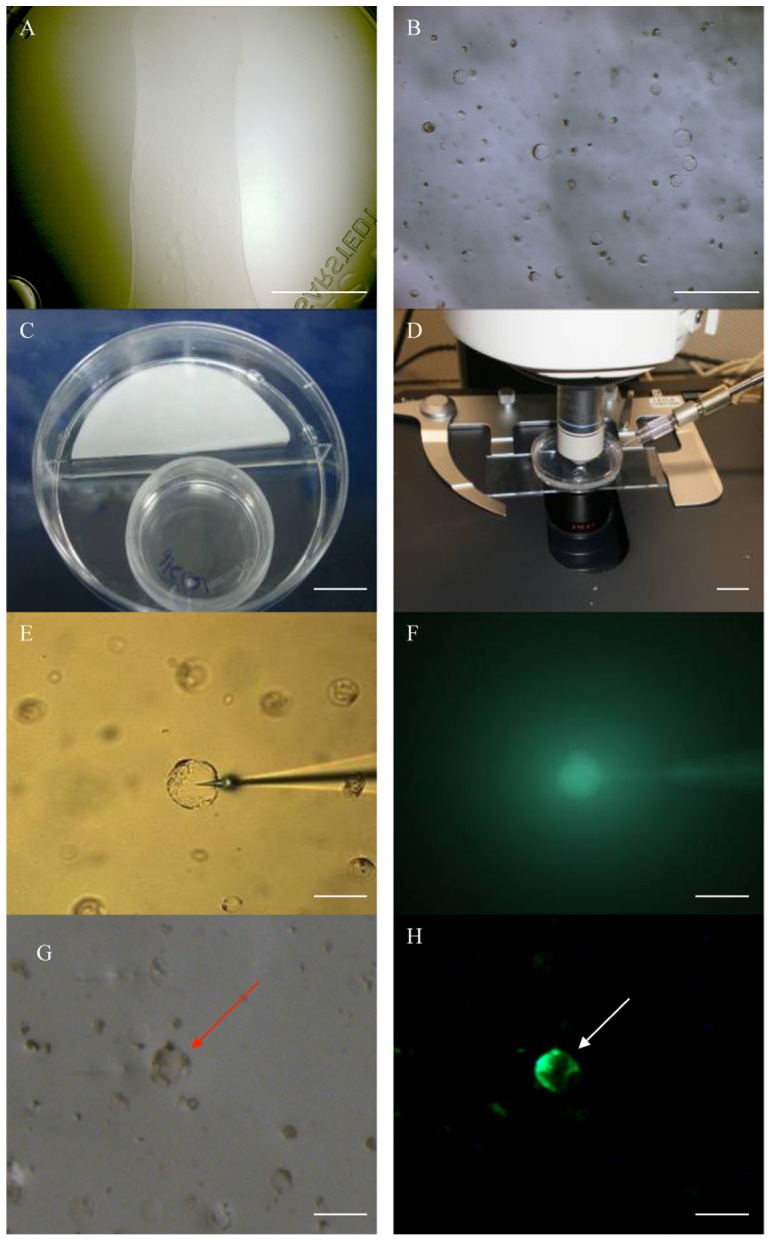
Microinjection of DNA into oil palm protoplasts. Oil palm protoplasts were isolated from a 3-month-old cell suspension culture after subculture for 7 days, mixed with 1% alginate solution in Y3A medium and distributed as a thin layer onto supporting medium (A). The embedded protoplasts were arranged in a single planar layer as confirmed by using the 10× objective (B). The protoplasts were incubated at 28°C in the dark for 3 days (C), and then placed on the microscope stage for DNA microinjection (D). The DNA solution was injected into the protoplast (E) and confirmed by Lucifer yellow fluorescence (F). GFP fluorescence was detected in the cytoplasm after 3 days (G and H). The injected protoplast is indicated by an arrow. Scale bar  = 1 cm in (A), (C) and (D), 100 µm in (B), 25 µm in (E)–(H).

A novel DNA microinjection protocol for oil palm protoplasts was developed using protoplasts embedded in an alginate layer (Figure 5A) because microinjection is facilitated if the protoplasts are immobilized in a single plane (Figure 5B). Different concentrations of alginate, ranging from 0.5% to 2%, were dissolved in Y3A liquid to prepare the substrate. We found that 1% alginate was ideal for immobilizing the protoplasts, whereas they remained mobile if lower concentrations were used and higher concentrations promoted the formation of clumps.

The embedded protoplasts were cultured for 3–4 days in a two-compartment dish (Figure 5C) allowing the partial development of the cell wall, which was the ideal time for DNA microinjection. Freshly-embedded protoplasts were damaged by the procedure, demonstrating that the fragile plasma membrane alone cannot withstand penetration by the needle tip. On the other hand, if the protoplasts were left for 5 or more days, the efficiency of microinjection was limited because the cell wall was by this stage fully developed. A single micromanipulator was used to inject all protoplasts because they were immobilized within the alginate layer (Figure 5D).

#### The injection of DNA and Lucifer yellow into the protoplast cytoplasm

The protoplasts were initially injected with the fluorescent dye Lucifer yellow in order to visualize the cytoplasmic compartment (Figure 5E–F). We then co-injected the dye and the linear CFDV-hrGFP fragment (Figure 5G–H). GFP expression was first detected 72 h after microinjection, and the GFP fluorescence was distributed throughout the cytoplasm and nucleus, extending to the plasma membrane. The emission wavelengths of Lucifer yellow and GFP are distinct, allowing us to clearly distinguish between cells expressing the microinjected DNA and those containing the marker dye alone.

#### The effect of DNA concentration on transformation efficiency, and the development of microcalli expressing GFP

**Figure 6:**
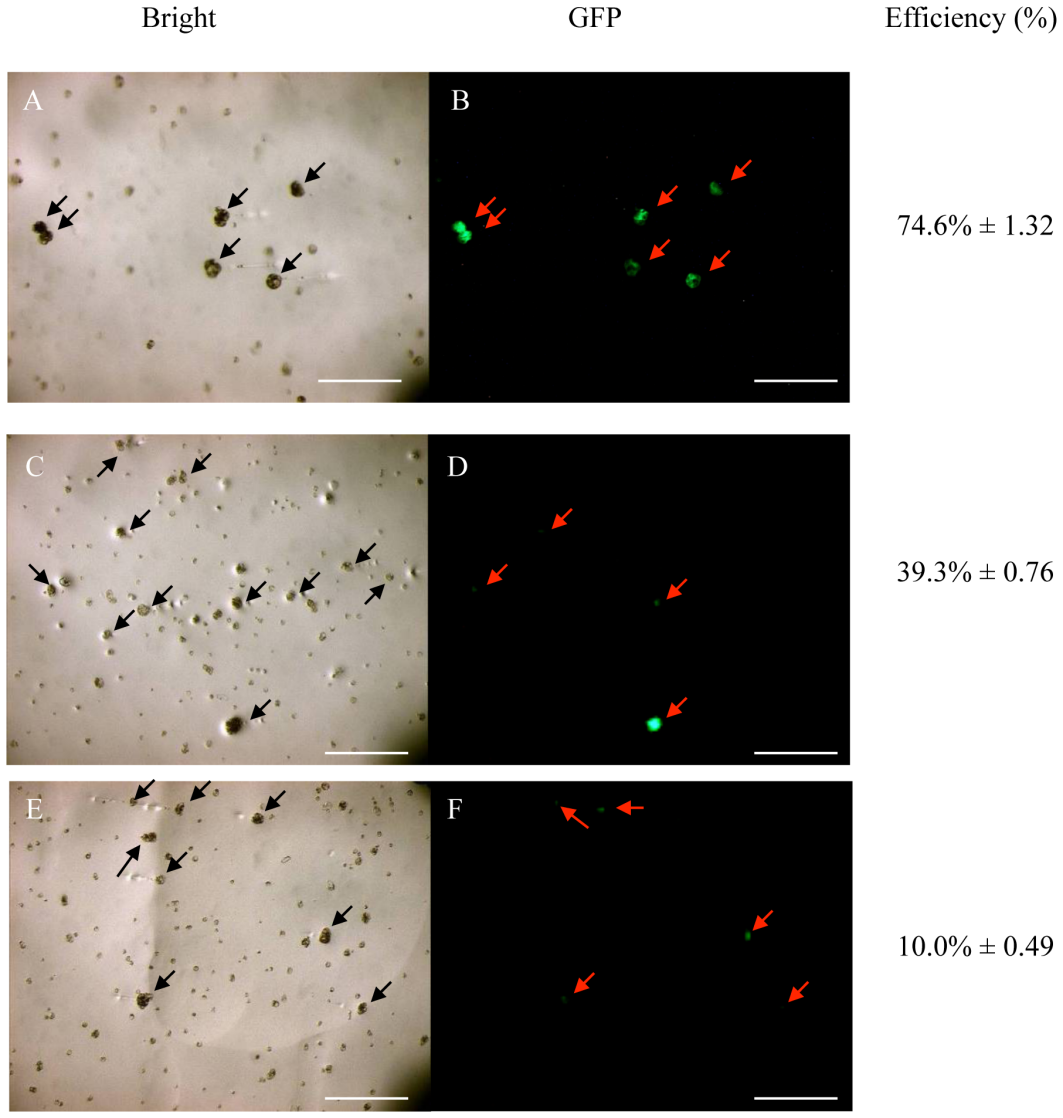
Effect of DNA concentration on transformation efficiency. Protoplasts were injected with 100/µl (A and B), 500 ng/µl (C and D) and 1000 ng/µl (E and F) DNA solutions and monitored for evidence of GFP fluorescence after culture for one month. The transformation efficiencies represent the mean of three replicates. Arrows indicate the surviving injected protoplasts and small dots indicate dead cells. Scale bar  = 100 µm. All cells were injected in the visial field shown in this figure, but uninjected cells also developed into (non-fluorescent) microcalli.

The optimal DNA fragment concentration was determined by comparing the transformation efficiencies achieved when injecting 50 embedded protoplasts with ∼5 µl of DNA solution at concentrations of 100 ng/µl (Figure 6A–B), 500 ng/µl (Figure 6C–D) and 1000 ng/µl (Figure 6E–F). After one month, we recorded corresponding transfection efficiencies of 74.6% (Figure 6A–B), 39.3% (Figure 6C–D) and 10% (Figure 6E–F), indicating that 100 ng/µl is the optimal concentration of microinjected DNA.

**Figure 7:**
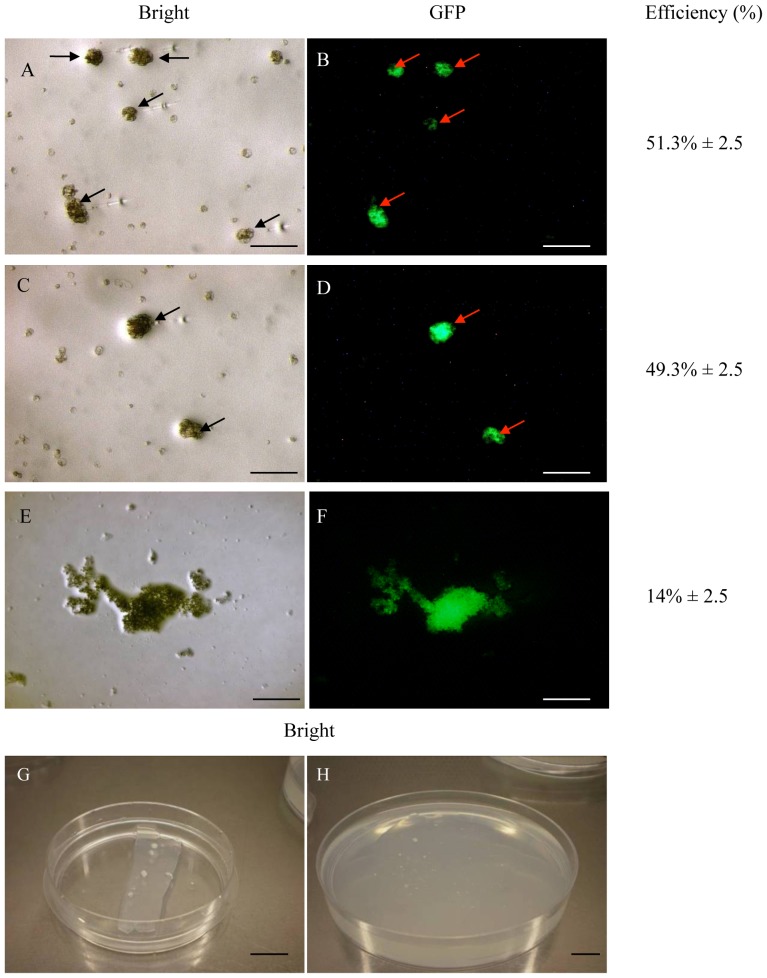
Development of microcalli expressing GFP. Five days after DNA microinjection, the alginate layer was transferred to Y3A liquid medium comprising 5.5% (w/v) sucrose and 8.2% (w/v) glucose supplemented with 10 µM NAA, 2 µM 2,4–D, 2 µM IBA, 2 µM GA_3_, 2 µM 2iP and 200 mg/l ascorbic acid and cultured at 28°C for 2 weeks. The medium was then replaced with similar Y3A liquid medium comprising 4% (w/v) sucrose and 7.2% (w/v) glucose to allow the development of microcolonies (A and B, after 2 months). The medium was then replaced with Y3A liquid medium comprising 4% (w/v) sucrose to promote the conversion of microcolonies (C and D, after 4 months) into microcalli (E and F, after 6 months). Finally, the alginate layer containing microcalli (G) was transferred onto Y31N0.1BA solid medium (H) for the regeneration of oil palm plants. Arrows indicate the injected protoplasts. The transformation efficiencies represent the mean of three replicates. Scale bar  = 100 µm in (A)–(F), 1 cm in (G) and (H).

Microcolonies developing from the protoplasts injected with 100 ng/µl DNA were observed for 2–3 months, by which time the proportion of colonies expressing GFP had fallen to 51.3% (Figure 7A–B). GFP expression was maintained for a further 2 months (Figure 7C–D) but the proportion of colonies expressing GFP fell to 14% after 6 months, when microcalli began to develop (Figure 7E–F). The microcalli expressing hrGFP were removed from the alginate layer (Figure 7G) and transferred to Y31N0.1BA solid medium (Figure 7H) for development into embryogenic calli, which is similar to the procedure for regenerating protoplasts into plants using agarose bead cultures [20]. A significant number of wild-type microcalli were also obtained in these experiments reflecting the absence of a selectable marker (data not shown).

## Discussion

### PEG-mediated transient expression in oil palm protoplasts

Genetic engineering in oil palm is challenging because the standard transformation approaches based on *Agrobacterium* and particle bombardment are laborious and inefficient, generating a large number of chimeric plants. Following the successful regeneration of oil palm plants from protoplasts derived from cell suspension cultures [20] it is now possible to use protoplasts as the starting material for the development of stable transgenic oil palm lines by genetic engineering. Protoplasts are beneficial as a starting material because they are totipotent, which allows transgenic plants to be regenerated from single cells thus avoiding the issue of chimeras. However, the transformation of oil palm protoplasts using *Agrobacterium* and particle bombardment is limited by the challenges described above, so novel transformation approaches are required in order to develop an efficient strategy for the generation of transgenic oil palm.

The stable integration of exogenous DNA into the genome of oil palm protoplasts following PEG-mediated transfection, electroporation or microinjection, could facilitate the generation of stable transgenic lines because the plants would be regenerated from a single transformed cell. However, these techniques have not been applied in oil palm before and the standard protocols would therefore need to be optimized to maintain the viability of oil palm protoplasts, promote the uptake of DNA and demonstrate the efficiency of transgene expression. Because PEG-mediated transfection is a standard method for gene transfer to protoplasts that allows the rapid analysis of transient reporter gene expression, this method was investigated as a first step in the development of an efficient transformation protocol.

We used GFP as a visual marker because this approach allows the recognition of transient expression just after gene transfer as well as stable expression after transgene integration in plant tissues (and whole plants) derived from transfected cells. In normal transformation approaches, selectable markers are required to allow the propagation of the rare, stably-transformed cells while killing or suppressing the large excess of non-transformed or transiently-transformed cells in the target tissue [21]. When single cells are the target, this selection process is unnecessary. However, the transfection of oil palm protoplasts using PEG is an untested strategy so it was necessary to provide evidence of transformation and regeneration efficiency, and for this purpose a visible marker such as GFP is ideal. Visual selection also allows transgenic plants to be developed without selectable markers, an approach which is considered increasingly attractive in a commercial setting [22].

Protoplasts isolated from oil palm suspension cultures after 7 days of subculture were identified as the most suitable substrates for PEG-mediated transformation. The protoplasts were uniform in size, and the transfected protoplasts were easily identified due to the absence of autofluorescence. In contrast, autofluorescence was observed in protoplasts from cell suspension cultures that were 3 and 4 months old after 14 days of subculture, which could produce false positive results. Protoplasts isolated from these cell suspension cultures should not have chloroplasts, so the autofluorescence may reflect the presence of small amounts of lipids. Osmotic stress during protoplast isolation can modulate lipid metabolism resulting in the synthesis of up to 27% palmitoleic acid [23].

The concentration of Mg^2+^ is the most important determinant of efficient PEG-mediated transient gene expression in tobacco and maize protoplasts [24], [25]. Similarly, we found that the concentration of Mg^2+^ greatly influenced the transfection efficiency of oil palm protoplasts and the intensity of GFP fluorescence. The highest transfection efficiency of 2.50% was achieved in the presence of 50 mM MgCl_2_ and the intensity of GFP fluorescence increased as the concentration of MgCl_2_ rose from 10 to 100 mM may indicating a more efficient uptake of the exogenous DNA and therefore the stronger expression of GFP, or a better cell survival, or both.

Incubation times with the exogenous DNA exceeding 10 min did not improve the transfection efficiency, and indeed reduced the proportion of cells expressing GFP by approximately four-fold (2.50% to 0.65%; compare Figure 2C to Figure 2E–F) suggesting that longer incubation times prolonged exposure to exogenous and endogenous nucleases, the former released from broken protoplasts. A slight improvement in transfection efficiency was therefore achieved with higher DNA concentrations (2.05% with 25 µg of DNA compared to 2.73% with 50 µg of DNA) because the larger amount of target DNA is likely to saturate the available nuclease pool.

The PEG-mediated transfection of oil palm protoplasts was also inhibited by the potentially toxic effects of excess PEG, resulting in a significant loss of viability in the presence of PEG concentrations exceeding 25% (w/v). High concentrations of PEG also promoted the clumping of protoplasts, making it difficult to identify individual protoplasts producing GFP. In contrast, a heat shock caused a significant improvement in transfection efficiency, probably by transiently permeabilizing the plasma membrane and thus promoting the uptake of DNA.

### Stable transformation of oil palm protoplasts by DNA microinjection

Initially, we microinjected DNA into protoplasts embedded in agarose beads, but although this method was successful it was also inefficient, allowing the injection of only 5–10 cells per hour (data not shown). The identification of protoplasts in this environment was often difficult, and the microinjection needle tips often became clogged with agarose debris after only 2–3 injection attempts. Furthermore, the preparation method required protoplasts to be exposed to molten liquid agarose at 45°C which imparted significant heat stress and reduced vitality. In contrast, protoplasts embedded in an alginate layer were ideal for DNA microinjection because alginate is transparent so the protoplasts could be identified easily, there was no heat stress during preparation so the protoplasts were more vital, and the flat surface made it easier to inject the protoplasts more rapidly (50–100 alginate embedded-protoplasts could be successfully injected per hour). Another advantage of the alginate layer was that it could be dissolved in sodium acetate solution to isolate the microcalli, allowing them to be transferred onto the appropriate media for further cultivation.

In previous studies, the nucleus of tobacco protoplasts was identified as the most suitable target compartment for DNA microinjection, with transformation frequencies of 14–20%, compared to 6% when the DNA was injected into the cytoplasm [26], [27]. However, we limited DNA microinjection to the cytoplasm because the nucleus was usually difficult to identify, and often became swollen and dislodged after injection resulting in the rapid death of the injected protoplasts (data not shown).

Based on the above experiments, the injection of ∼5 µl of DNA (at concentration of 100 ng/µl) into the cytoplasm of protoplasts embedded in an alginate layer was identified as the optimal platform for the transformation of oil palm protoplasts. This resulted in approximately 14% of the injected protoplasts developing into microcalli that continued to express GFP. Although this is the first report of genetic engineering in oil palm by DNA microinjection, the transformation efficiency of 14% is far higher than that achieved using other approaches such as PEG-mediated transfection (4.76%), particle bombardment (1%) [3] and *Agrobacterium*-mediated transformation (0.7%) [17]. Our novel microinjection approach in oil palm therefore represents a significant increase in the efficiency of transformation in this species.

### Advantages of DNA microinjection for the genetic engineering of oil palm

Particle bombardment and *Agrobacterium*-mediated transformation are the most widely used procedures for genetic engineering in plants and both have been applied to oil palm, but in each case the techniques are inefficient and beset by additional disadvantages. The novel and highly efficient transformation approaches we have developed for oil palm protoplasts, i.e. PEG-mediated transfection and particularly DNA microinjection, offer new routes to overcome these barriers and improve the efficiency and applicability of genetic engineering in this erstwhile recalcitrant species.

Both particle bombardment and *Agrobacterium*-mediated transformation require at least 0.5 g of target tissue for each transformation experiment, which involves laborious and time-consuming preparation. In contrast, less than 0.5 g of oil palm tissue is required for protoplast isolation, and each explant yields thousands of protoplasts for subsequent transformation experiments. Another advantages is that ‘clean gene’ fragments consisting of only the promoter-gene-terminator sequence can be introduced by microinjection into the oil palm protoplast, thereby avoiding the integration of vector backbone sequences that can interfere with transgene expression and raise concerns with the regulatory authorities [22].

The genetic engineering of oil palm protoplasts by microinjection could allow the production of stable and non-chimeric transgenic lines regenerated from a single transformed cell, each carrying a single copy of the integrated transgene (which is an important consideration for the commercialization of transgenic oil palm plants) [27]. Nevertheless, further improvements are required before this approach becomes a standard technique in oil palm transformation programs. For example, the current transformation efficiency of 14% is relatively low compared to the frequencies achieved in other species, such as 26% in alfalfa [28] and 20–53% in tobacco [27], [29].

## Materials and Methods

### Plant material

Oil palm embryogenic cell suspension cultures were cultivated in 100-ml flasks containing 50 ml Y35N5D2iP liquid medium [20] supplemented with 5 µM naphthalene acetic acid (NAA), 5 µM 2,4-dichlorophenoxyacetic acid (2,4-D) and 2 µM 2-γ-dimethylallylaminopurine (2iP). The suspension cultures were incubated in the dark at 28°C on a rotary shaker at 120 rpm. Half of the Y35N5D2iP liquid medium was discarded and replaced with fresh medium every 14 days.

### Protoplast isolation and purification

Protoplasts were isolated from 3-month-old and 4-month-old oil palm cell suspension cultures. The cells were collected by filtration through a 300-µm nylon mesh, and 0.5 g fresh weight (fwt) of cells was transferred to a 50-ml centrifuge tube containing 15 ml filter-sterilized enzyme solution (2% (v/v) cellulase (Sigma), 1% (v/v) pectinase (Sigma), 0.5% (w/v) cellulase onuzuka R10 (Duchefa), 0.1% (w/v) pectolyase Y23 (Duchefa), 3% (w/v) KCl, 0.5% (w/v) CaCl_2_.2H_2_O and 3.6% (w/v) mannitol, pH 5.6). The cells were resuspended by inverting the tube 6–10 times and then incubated in the dark without shaking at 26°C for 14 h. The mixture was diluted with 15ml filter-sterilized washing solution (3% (w/v) KCl, 0.5% (w/v) CaCl_2_.2H_2_O, 3.6% (w/v) mannitol, pH 5.6), resuspended by inverting the tube 3–5 times, filtered through a sterilized double layer of miracloth and collected in a 50-ml centrifuge tube. The filtration step was repeated 2–3 times until all undigested tissues, cell clumps and cell debris were removed. The mixture was centrifuged at 60×g for 5 min at 22°C and the supernatant was discarded. The protoplast pellet was resuspended by adding 10 ml washing solution and mixing by inversion, followed by centrifugation as above. The supernatant was removed completely and the protoplast pellet was resuspended in 10 ml filter-sterilized rinse solution (3% (w/v) KCl, 3.6% (w/v) mannitol, pH 5.6) and then centrifuged at 60×g for 5 minutes at 22°C. After three cycles, the supernatant was removed leaving 3 ml in the tube, and was stored at room temperature for further experiments.

### PEG-mediated transfection

A 1-ml aliquot of the protoplast suspension was incubated at room temperature for 10 min, or a heat shock was applied by incubation at 45°C for 5 min before immediately cooling on ice for 1 min, and then incubated at room temperature for 10 min. A 500-µl aliquot of the protoplast suspension was then placed as a single droplet in the middle of a 60 mm×15 mm Petri dish (Greiner Bio-One, Germany) and five drops of 100 µl PEG-MgCl_2_ solution (25–50% (w/v) PEG 4000 (Sigma), 10–100 µM MgCl_2_ (Sigma), 3% (w/v) KCl, 3.6% (w/v) mannitol, 0.05% (w/v) 2-N-morpholinoethanesulfonic acid (MES), pH 6.0) were added in an adjacent but separate position. We then slowly added 25 or 50 µg of plasmid DNA to the protoplasts and mixed gently by stirring with a 200-µl pipette tip. The mixture was incubated at room temperature in the dark for 10–30 min and then the protoplasts + DNA were mixed with the adjacent PEG/MgCl_2_ drops by stirring with the 200-µl pipette tip. After a further 30-min incubation, 4 ml of washing solution (3% (w/v) KCl, 0.5% (w/v) CaCl_2_.2H_2_O, 3.6% (w/v) mannitol, pH 5.6) was added drop by drop and the mixture was incubated in the dark at 26°C for 9 days. The protoplasts were observed under a Leica TCS 5 SP5 X confocal laser scanning microscope (CLSM) and visualized using a Leica Microsystem LAS AF. GFP fluorescence was observed at an excitation wavelength of 488 nm and an emission range of 500–600 nm, whereas autofluorescence of protoplasts was excited at 543 nm and detected within the emission range 675–741 nm. PEG-mediated transfection efficiency was calculated as the number of GFP-positive protoplasts divided by the total number of protoplasts in one representative microscope field. Each calculation was carried out for a total of three microscope fields containing no fewer than 200 protoplasts.

### Preparation of alginate thin layer

After allowing the protoplast suspension to settle for 30 min, the supernatant was removed completely and the pellet was resuspended in 3 ml filter-sterilized alginate solution comprising 1% (w/v) alginic acid sodium salt (A2158, Sigma) dissolved in Y3A liquid medium [20] (5.5% (w/v) sucrose, 11.9% (w/v) glucose, 10 µM NAA, 2 µM 2,4-D, 2 µM IBA, 2 µM GA_3_, 2 µM 2iP and 200 mg/l ascorbic acid) adjusted to pH 5.6, and including Y3 macroelements prepared without CaCl_2_. Alginate-embedded protoplasts were distributed as a thin layer onto a substrate comprising 1.5 ml filter-sterilized Y3A medium (5.5% (w/v) sucrose and 11.9% (w/v) glucose supplemented with 0.1% (w/v) CaCl_2_.2H_2_O and solidified with 1% (w/v) agarose sea plaque) in a 35×10 mm Petri dish (Greiner Bio-One, Germany). The distribution of alginate-embedded protoplasts was achieved by dropping 100 µl alginate-embedded protoplasts at the edge of the Petri dish and tilting to 35° so the drop was distributed as a thin layer. The dishes were placed horizontally into 94×15 mm two-compartment dishes (Greiner Bio-One, Germany) allowing the alginate to solidify within 1–2 min. We added 3 ml of sterile water to the outer compartment to prevent the alginate layer from drying out. The plates were sealed and incubated at 28°C in the dark for 3 days.

### Microinjection workstation

The microinjection workstation consisted of a Leica DM LFS upright microscope (Leica Microsystems Wetzlar GmbH, Germany) with a joystick-controlled motorized objective revolver for HCX APOL U-V-I water immersion objectives (10x, 20x, 40x and 63x) mounted on a fixed table and placed under a laminar flow hood. The microscope was equipped with a Luigs and Neumann Manipulator set with a control system SM-5 and SM-6 (Luigs and Neumann, Germany).

### Preparation of DNA injection solution

Plasmid DNA was prepared using a midi-scale Plasmid DNA Purification Kit (NucleoBond PC100; MACHEREY-NAGEL, Germany) and was dissolved at concentration of 1 µg/µl in sterile water. The plasmid was digested with HindIII and EcoRI to yield the CFDV-hrGFP-nos cassette as a 1.5-kb fragment. The fragment was separated from the vector sequence (pUC19) by 1% agarose gel electrophoresis, excised using a clean blade and isolated using the PCR clean-up Gel Extraction Kit (NucleoSpin Gel and PCR Clean-up) according to the manufacturer's instructions (Macherey-Nagel, Germany). The DNA cassette was then diluted in sterile water to concentrations of 100 ng/µl, mixed at a 100∶1 ratio with Lucifer yellow CH dilithium salt (L0259, Invitrogen) and filter-sterilized using the Ultafree-MC filter (Durapore 0.22 µm, type GV; SK-1M-524-J8; Millipore) at 10,000 rpm, 15 min, 4°C. The eluted DNA was partitioned into 10 µl aliquots and stored at −20°C.

### Loading the DNA injection solution into microinjection needle

The DNA injection solution was centrifuged at 14,000 rpm for 30 min at 4°C and a 5-µl aliquot was loaded into the tip of a Femtotip II microinjection needle (no. 5242 957000, Eppendorf) using a microloader (no. 5242 956003, Eppendorf). After 30 min at room temperature, the needle was filled with sterile mineral oil (M8410, Sigma) using the microloader and tightly mounted in the capillary holder of a microinjector CellTram vario (no. 5176 000033, Eppendorf), and then fixed onto the micromanipulator.

### Microinjection of oil palm protoplasts

A plate containing protoplasts embedded in an alginate layer was placed on the microscope stage, and the vitality of the protoplasts was confirmed using the 10× objective, which was then raised to its maximum height allowing the needle tip to reach the center of the field view freely with the X/Y-axis controller (Control system SM-5) of the manipulator. The needle was lowered as close as possible to the alginate layer with the Z-axis controller and the cytoplasm or nucleus of the target protoplast was identified by adjusting the 20× objective to optimal resolution and contrast. The needle tip was then moved to immediately above the protoplast with the X/Y-axis controller and inserted into the alginate layer immediately adjacent to the protoplast using the Z-axis controller before penetration using the X-axis controller. The DNA solution was slowly injected into the protoplast using a microinjector CellTram vario, and confirmed by fluorescence illumination. The needle tip was carefully withdrawn from the protoplast and moved to the next one. The injected protoplasts were monitored periodically using a Leica MZ16F fluorescent stereomicroscope with a GFP3 filter (Leica Microsystems Wetzlar GmbH, Germany).

### Alginate layer culture

Following microinjection, the plates containing the alginate layer were incubated in the dark at 28°C for 5 days. The alginate layers were then separated from the substrate and transferred into 60×15 mm Petri dishes containing 3 ml Y3A liquid medium comprising 5.5% (w/v) sucrose and 8.2% (w/v) glucose supplemented with 10 µM NAA, 2 µM 2,4-D, 2 µM IBA, 2 µM GA_3_, 2 µM 2iP and 200 mg/l ascorbic acid. The dishes were incubated in the dark at 28°C, shaking at 50 rpm. After 2 weeks, the medium was replaced with similar Y3A liquid medium but the concentrations of sucrose and glucose were reduced to 4% (w/v) and 7.2% (w/v), respectively. The alginate layers were cultured in this medium for one month by refreshing the medium at 14-day intervals, then replaced with Y3A liquid medium containing 4% (w/v) sucrose until microcalli had developed.
